# LUMBAR FACET ARTHROPLASTY FOR SPONDYLOLISTHESIS SURGICAL CORRECTION: SUMMARY OF EVIDENCES

**DOI:** 10.1590/1413-785220253303e287254

**Published:** 2025-08-18

**Authors:** Rafael Augusto da Silva Aparício, Felipe de Almeida Pinto, Rafael Félix Alves, Victor Hugo Cordeiro Ribeiro

**Affiliations:** 1 Hospital Geral de Carapicuiba, Sao Paulo, SP, Brazil.; 2 Santa Casa de Misericordia de Ribeirao Preto, Ribeirao Preto, Sao Paulo, SP, Brazil.

**Keywords:** Orthopedics, Spine, Spondylolisthesis, Arthroplasty, Ortopedia, Coluna, Espondilolistese, Artroplastia

## Abstract

In persistent and debilitating cases of spondylolisthesis, surgical correction is usually indicated, and the surgeon may opt for spinal fusion or dynamic stabilization techniques, such as lumbar facet arthroplasty (LFA). However, LFA is an emerging technique that requires more research to fully understand its effectiveness and safety. To summarize the scientific evidence related to LFA in the surgical correction of spondylolisthesis. The database chosen to papers selection was PUBMED, based on the following search string: ((lumbar[title/abstract] AND facet[title/abstract] AND arthroplasty[title/abstract]) OR tops[title /abstract]) AND spondylolisthesis[title/abstract], including studies published in the last 10 years. Results: Initially, 9 papers were identified that met the previously established search strategy. After reading the titles and abstracts, 1 text was excluded because it did not deal with the application of LFA in spondylolisthesis, leaving 8 studies for review. Significant clinical improvement, maintenance of radiological stability and low complication rates were consistently observed with the use of LFA. Furthermore, economic analyzes indicated that the TOPS System is a cost-effective and economically dominant option compared to conventional approaches such as TLIF. These findings suggest that the TOPS system may be a viable and advantageous alternative for patients with degenerative spondylolisthesis, offering not only clinical but also long-term economic benefits. ***Level of Evidence IV; Descriptive Studies***.

## RESUMO

Em casos persistentes e debilitantes de espondilolistese costuma-se indicar a correção cirúrgica, podendo o cirurgião optar pela fusão espinal ou por técnicas de estabilização dinâmica, como a artroplastia facetária lombar (AFL). Todavia, a AFL é uma técnica emergente que requer mais pesquisas que visem entender completamente sua eficácia e segurança. Sintetizar as evidências científicas relacionadas à AFL na correção cirúrgica da espondilolistese. A base de dados escolhida para seleção dos trabalhos foi a PUBMED, com base na seguinte string de busca: ((lumbar[title/abstract] AND facet[title/abstract] AND arthroplasty[title/abstract]) OR tops[title/abstract]) AND spondylolisthesis[title/abstract], incluindo artigos publicados nos últimos 10 anos. Inicialmente foram identificados 9 trabalhos que atenderam a estratégia de busca previamente estabelecida. Após a leitura dos títulos e resumos, 1 texto foi excluído por não tratar da aplicação da AFL na espondilolistese, restando 8 trabalhos para revisão. Observou-se consistentemente uma melhora clínica significativa, manutenção da estabilidade radiológica e baixas taxas de complicações com o uso da AFL. Além disso, análises econômicas indicaram que o Sistema TOPS é uma opção custo-efetiva e economicamente dominante em comparação com as abordagens convencionais, como a TLIF. Esses achados sugerem que o sistema TOPS pode ser uma alternativa viável e vantajosa para pacientes com espondilolistese degenerativa, oferecendo não apenas benefícios clínicos, mas também econômicos a longo prazo. ***Nível de Evidência IV; Estudos Descritivos***.

## INTRODUCTION

Espondylolisthesis is a term widely used to describe the anterior, lateral or posterior sliding of one vertebral body over another.^1^ The presentation of espondylolisthesis can vary widely, including, but not limited to, compressive neurological defects (spinal stenosis), mild to intense lumbalgia, as well as aesthetic problems.^1,2^

Although many patients respond to conservative treatment (non-steroidal anti-inflammatory agents - NSAIDs, orthoses, etc.), many cases often require decompression, fusion, reduction, fixation, among other surgical interventions.^1,3^ The main indication for surgical treatment of patients with spondylolisthesis are persistent and debilitating symptoms that did not respond to conservative treatment.^4^

The vast majority of patients with spondylolisthesis are asymptomatic. However, when present, the symptoms usually derive from mechanical etiology or spinal stenosis, and the individuals often complain of intermittent neurogenic claudication, a consequence of spinal stenosis that presents as lumbar pain with irradiation to the proximal bilateral lower extremities, with paresthesia and weakness associated with deambular or orthostaticism.^5,6^

Although the treatment of spondylolisthesis generally tends to be more conservative in an effort to minimize risk and maximize results, surgical treatment should be considered in patients with persistent and debilitating symptoms.^7^ Dynamic stabilization is an alternative that minimizes degeneration from the adjacent level, restoring segmental stability while retaining movement in the affected segment.^8^ Lumbal facet arthroplasty (LFA) is a proposed method of dynamic stabilization intended to treat stage I spondylolisthesis with stenosis.^9^ The procedure is performed under general anesthesia, and involves surgical incision in the lumbar area for exposure of affected facetary joints. The damaged structures are removed and replaced with artificial implants (TOPS - Total Posterior Spine System) designed to restore proper biomecany and segmental stability.^9,10^ Within this context, considering LFA as a relatively recent surgical approach, the procedure has aroused growing interest in the medical and scientific community. However, due to its innovative nature, the quality and consistency of the available evidence can be variable, presenting gaps and inconsistencies that require clarification. Therefore, a comprehensive summary of the publications on the subject is necessary to clarify the efficacy, safety and precise indications of this surgical technique. Such results would contribute not only to improving the understanding of the clinical and functional outcomes associated with LFA, but would also serve the north for the improvement of clinical practices and therapeutic decision-making in front of patients with spondylolisthesis.

## MATERIAL AND METHOD

This is an exploratory study that was based on the method of integrative review of literature with evidence synthesis. According to the proposed by Souza et al.^11^, the integrative review should follow six steps for its preparation, which are: 1 - definition of the question that guided the review; 2 - identification in the literature of the works relevant to the topic and already published; 3 - collection of data on the specified basis; 4 - thorough and critical analysis of the selected studies; 5 - discussion of the selected results; and 6 - preparation and presentation of the integrative review. Initially, the research question that guided this review was the following: "What are the clinical evidence related to the execution of lumbar facetary arthroplasty for surgical correction of spondylolisthesis?". For the second stage of the review, i.e., the selection of the articles to be reviewed, the database chosen for the selection of the papers was PUBMED, using the following search strategy: *((lumbar[title/abstract] AND facet[title/abstract] AND arthroplasty[title/abstract]) OR tops[title/abstract]) AND spondylolisthesis[title/abstract]*. Articles published between January 2014 and April 2024 were selected and all papers discussing the topic were initially considered for inclusion in the sample. After sorting the titles and summaries, studies that did not address the use of LFA in the surgical correction of spondylolisthesis were excluded from the analysis.

## RESULTS

The third phase of the integrative review, which concerns the collection and analysis of the information identified in the database, took place in May 2023. Initially 9 jobs were identified that met the previously established search strategy. After reading the titles and summaries, one text was deleted for not dealing with the application of LFA in spondylolisthesis, remaining 8 papers for review. At this stage, the selected articles were read in their entirety, summarized, and presented in the following section ordered according to the year in which they were published.

## Discussions

Anekstein et al.,^12^ evaluated the clinical feasibility and improvement provided by the TOPS System in the surgical treatment of lumbar degenerative spondylolisthesis and/or spinal stenosis. For this purpose, over a period of one year (June 2006 to July 2007), ten patients were included in a prospective, non-randomized clinical trial. The primary indication was neurogenic claudication due to spinal stenosis with single-level degenerative spondylolisthesis. As a result, the VAS score for back pain fell from 56.2 in preoperative to 12.5 in six weeks and 19 in seven years of follow-up. The VAS score for worse leg pain fell from 83.5 before surgery to 13 to six weeks and 8.8 to seven years of follow-up. The ODI fell from 49.1 in preoperative to 13.5 in six weeks and 7.8 in seven years of follow-up. The MRI examination seven years after the surgery showed no adjacent stenosis to the stabilized segment and spondylolisthesis did not progress in either case. One patient experienced symptomatic lateral disc hernia (L3-L4) five years after surgery, whose symptoms were resolved with non-operative treatment. In one patient, conversion to post-lateral fusion was carried out due to early malfunction of the device. For the authors, in patients with spinal stenosis and degenerative spondylolisthesis, decompression and posterior artroplasty with the TOPS system could maintain clinical improvement and radiological stability over time.

Smorgick and collaborators,^13^ evaluated the long-term clinical outcome of the TOPS System in the surgical treatment of lumbar spinal stenosis with degenerative spondylolisthesis. Therefore, between June 2006 and July 2007, 10 patients with neurogenic claudication due to spinal stenosis and single-level degenerative spondylolisthesis were included in a prospective, non-randomized clinical study. The average VAS score for leg pain fell from 83.5 before surgery to 13 to 6 weeks and 17 to 11 years after surgery. The average VAS score for back pain fell from 56.2 in preoperative to 12.5 in six weeks and 14 in 11 years after surgery. The average ODI score decreased from 49.1 in preoperative to 13.5 in six weeks and 16 in 11 years after surgery. MRI at the age of 11 demonstrated stenosis adjacent to the stabilized segment in a patient, but the same was not symptomatic. The authors found no evidence of progression of spondylolisthesis in either case. In one patient, conversion to post-lateral fusion was carried out due to early malfunction of the device. For the authors, the results of this 11-year follow-up study showed that, in patients with spinal stenosis and degenerative spondylolisthesis, decompression and LFA maintain clinical improvement and radiological stability.

Haleem et al.,^14^ through a feasibility study, evaluated the five-year mean results in patients treated with the facetary replacement system TOPS in the surgical treatment of lumbar spinal stenosis and degenerative spondylolisthesis. For this, 10 patients (two men, eight women, average age: 59,6 years) were included in a prospective, non-randomized clinical study. It was noted that the scores of the clinical results of the cohort improved significantly in all scoring systems. The two-year-old X-rays revealed no loss of position or loosening of metalwork. There were two incidental durotomies and no failures in five years, with no patient requiring revision surgery. For the authors, the TOPS System retained the clinical improvement and movement in the surgical management of lumbar spinal stenosis and spondylolisthesis, suggesting that it could be considered an option for these indications.

Ament and collaborators,^10^ comprehensively evaluated the cost-benefit ratio of the TOPS System in comparison with the control group, which underwent a standard lumbar transforaminal intersomatic fusion (TLIF). With this, the researchers aimed to evaluate the cost-effectiveness ratio of TOPS compared to TLIF. The laboratory patient population was extracted from a multicenter randomized clinical trial (n = 121) with full follow-up of one year. The results showed that from the healthcare system point of view, assuming a 50/50 division between Medicare and private payers, the TOPS cohort was cost-effective two years after surgery (US$6.158/QALY) compared to the control. At six years or older, TOPS became dominant, regardless of the mix of payers and the surgical environment. With $100,000/QALY payable availability limits, 63% of all 5,000 input parameter simulations favoured the TOPS System, even with an increase of $4,000 compared to TLIF. For the authors, the TOPS System was profitable compared to the TLIF and became the dominant economic strategy over time. Lack et al.,^15^ evaluated the experience of a single center with a facetary replacement implant (TOPS System) designed to stabilize the spine and prevent further degeneration while keeping a normal range of motion (ROM). For this purpose, 17 patients with lordosis and/or degenerative spondylolisthesis received the implant after laminotomy. It was identified that the average operating time was 102 minutes and the average reduction in VAS was 7.5 to 3 months, 6.8 to 12 months and 6.7 in the longest follow-up (average: 51 months, range: 26-77), showing an average improvement of 81%. The average pre- and post-operative ROM was 8.2° and 7.4°, respectively. For the authors, this series showed that the TOPS System has the potential to relieve back pain and keep ROM close to normal for long periods of time without inducing adjacent segment diseases.

Coric and colleagues,^16^, evaluated the safety and efficacy of a rear facet replacement device, the TOPS System, for the treatment of symptomatic lumbar stenosis of a level with Degree I degenerative spondylolisthesis. For this, they conducted a prospective, randomized and controlled study comparing the TOPS System with TLIF and fixation with pedicular screw. The minimum duration of follow-up was 24 months and the measurements of validated outcomes reported by patients included ODI and VAS for back and leg pain. 249 patients were evaluated (n = 170 in the TOPS group and n = 79 in the TLIF group), and there were no statistically significant differences between implanted levels or blood loss. The overall composite measure of clinical success was significantly higher in the TOPS group (85%) compared to the TLIF group (64%). The percentage of patients reporting a minimum 15 point improvement in ODI showed a statistically significant difference favouring TOPS (93%) compared to TLIF (81%). There was no statistically significant difference between the groups in the percentage of patients reporting a minimum 20 points improvement in VAS scores for back pain (TOPS, 87%; TLIF, 64%) and leg pain (TOPS, 90%; TLIF, 88%). The rate of surgical reintervention for facetary replacement in the TOPS group (5.9%) was lower than in the TLIF group (8.8%) and the TOPS cohort demonstrated maintenance of flexion/extension ROM from preoperative (3.85°) to 24 months (3.86°). For the authors, this study demonstrated that posterior lumbar decompression and dynamic stabilization with the TOPS System are safe and effective in the treatment of lumbar stenosis with degenerative spondylolisthesis. In addition, the decompression and dynamic stabilization with the TOPS System kept the movement segmented.

Pinter et al.,^9^ through a randomized prospective clinical trial, reported clinical and radiographic results in one year and the safety profile of patients undergoing LFA through the implantation of the TOPS System. In total, 153 patients were evaluated. The mean surgical time was 187.8 minutes, the estimated average blood loss was 205.7 cc and the average hospitalization time was 3 days. The mean scores of ODI, VAS for leg and back pain and the Zurich Claudication questionnaire improved significantly at all postoperative times. There were no clinically significant changes in radiographic parameters and all surgical segments remained mobile during one-year follow-up. Postoperative complications occurred in 11 of the 153 patients (7.2%) submitted to the TOPS device implant. Nine patients (5.9%) underwent a total of 13 re-operations, one (0.6%) for device-related failure due to bilateral release of the pedicular screw L5. For the authors, LFA with the TOPS System demonstrated a statistically significant improvement in all measurements of patient-reported results and in the ability to maintain movement at the level of the index, limiting sagital translation with a low rate of complications.

Finally, Ament and collaborators,^17^ evaluated the cost-benefit ratio of the TOPS System compared to the TLIF based on an updated data set from the FDA. For this purpose, they conducted a cost-utility analysis of the TOPS system compared to TLIF through a multicenter randomized clinical trial that investigated the effectiveness of the two modalities in a population of 305 enrolled, with 168 patients being followed for two years. The updated base case result demonstrated that the TOPS was immediate and longitudinally dominant compared to the control, with an incremental cost-effectiveness ratio of US$9,637,37/QALY. The net monetary benefit was $2,237, both in the perspective of the healthcare system and at a DAP threshold of $50,000/QALY over the two-year period. This remained true in all the tested scenarios. The Sensitivity Analysis of Alternative Scenarios suggested cost-benefit regardless of the type of payer and the surgical environment and to maintain the good cost-benefit ratio, the cost difference between TOPS and TLIF should not exceed US$1,875 and US$3,750 in DAP limits of US$50,000 and US$100,000/QALY, respectively. For the authors, the analysis confirmed that the TOPS System was a surgical treatment option with a good cost-benefit ratio and economically dominant for patients with lumbar stenosis and degenerative spondylolisthesis compared to TLIF in all the examined scenarios.

## CONCLUSION

In this final stage of the work, the presentation of the integrative review itself, synthesizing its main evidence, and considering the various studies evaluated on the efficacy and safety of LFA using the TOPS System in the treatment of degenerative spondylolisthesis, a consistent clinical improvement, maintenance of radiological stability and low complication rates with the use of LFA were observed. In addition, economic analyses have indicated that the TOPS System is a cost-effective and economically dominant option compared to conventional approaches such as TLIF. These findings suggest that the TOPS system may be a viable and advantageous alternative for patients with degenerative spondylolisthesis, offering not only clinical but also long-term economic benefits.
